# Previous Extrapulmonary Malignancies Impact Outcomes in Patients With Surgically Resected Lung Cancer

**DOI:** 10.3389/fsurg.2021.747249

**Published:** 2021-10-05

**Authors:** Hsin-Ying Lee, Min-Shu Hsieh, Hsien-Chi Liao, Pei-Hsing Chen, Xu-Heng Chiang, Kuan-Chuan Tsou, Tung-Ming Tsai, Jen-Hao Chuang, Mong-Wei Lin, Hsao-Hsun Hsu, Jin-Shing Chen

**Affiliations:** ^1^Department of Medicine, National Taiwan University, Taipei, Taiwan; ^2^Department of Pathology, National Taiwan University Hospital and National Taiwan University College of Medicine, Taipei, Taiwan; ^3^Department of Traumatology, National Taiwan University Hospital and National Taiwan University College of Medicine, Taipei, Taiwan; ^4^Department of Surgery, National Taiwan University Hospital Yun-Lin Branch, Douliu, Taiwan; ^5^Department of Surgery, Taipei City Hospital, Taipei, Taiwan; ^6^Department of Surgical Oncology, National Taiwan University Cancer Center, Taipei, Taiwan; ^7^Department of Surgery, National Taiwan University Hospital and National Taiwan University College of Medicine, Taipei, Taiwan

**Keywords:** clinicopathological feature, extrapulmonary malignancy, overall survival, prognosis, breast cancer, lung resection

## Abstract

**Background:** As the overall survival of patients with cancer continues to improve, the incidence of second primary malignancies seems to be increasing. Previous studies have shown controversial results regarding the survival of patients with primary lung cancer with previous extrapulmonary malignancies. This study aimed to determine the clinical picture and outcomes of this particular subgroup of patients.

**Materials and Methods:** We included 2,408 patients who underwent pulmonary resection for primary lung cancer at our institute between January 1, 2011 and December 30, 2017 in this retrospective study. Medical records were extracted and clinicopathological parameters and postoperative prognoses were compared between patients with lung cancer with and without previous extrapulmonary malignancies.

**Results:** There were 200 (8.3%) patients with previous extrapulmonary malignancies. Breast cancer (30.5%), gastrointestinal cancer (17%), and thyroid cancer (9%) were the most common previous extrapulmonary malignancies. Age, sex, a family history of lung cancer, and preoperative carcinoembryonic antigen levels were significantly different between the two groups. Patients with previous breast or thyroid cancer had significantly better overall survival than those without previous malignancies. Conversely, patients with other previous extrapulmonary malignancies had significantly poorer overall survival (*p* < 0.001). The interval between the two cancer diagnoses did not significantly correlate with clinical outcome.

**Conclusion:** Although overall survival was lower in patients with previous extrapulmonary malignancies, previous breast or thyroid cancer did not increase mortality. Our findings may help surgeons to predict prognosis in this subgroup of patients with primary lung cancer.

## Introduction

In recent decades, major improvements in cancer treatment, including molecular targeted and immune modulation therapies, have allowed patients to survive long enough to develop subsequent primary malignancies (1). Moreover, advancements in diagnostic approaches, including new imaging techniques and cancer biomarkers, have resulted in earlier and higher detection rates of multiple primary cancers (2, 3). Defined as cancers with more than one independent primary malignancy in the same or different organs, multiple primary cancers may develop in a synchronous or metachronous fashion (4). Although this phenomenon was first described by Billroth (5) in 1889, Cahan et al. (6) in 1969 were the first to report multiple primary cancers involving the lungs. Later, Hofmann et al. (7) demonstrated the incidence of second primary lung cancer at 1.6 per 100,000 population. More recently, Hu et al. (8) identified 178 (5.0%) patients with primary lung cancer undergoing surgery who had different types of previous extrapulmonary malignancies.

While there is no doubt that multiple primary malignancies involving the lungs can occur, the therapeutic strategies and survival outcomes of patients with lung cancer with a history of extrapulmonary malignancies remain controversial. An increased risk of synchronous lung cancer in patients with breast cancer has been reported, suggesting that genetic factors may increase the risk of multiple primary malignancies (9). Additionally, the widespread use of radiotherapy and common risk factors may contribute to an increased risk of second primary lung cancer in patients with a history of head and neck cancer (10–12).

In this study, we aimed to analyze the survival differences among patients with lung cancer with and without previous extrapulmonary malignancies. Additionally, we sought to identify potential risk factors for second primary lung cancer occurrence to develop strategies aimed at prevention and early diagnosis among high-risk individuals.

## Materials and Methods

### Study Population

In this retrospective study, we obtained data from 2,737 patients with newly diagnosed primary lung cancer who underwent pulmonary resection performed by a single surgical team at the National Taiwan University Hospital between January 2011 and December 2017. A history of previous extrapulmonary malignancies was histologically confirmed from patients' medical records. Controversial cases with possible lung metastases were excluded to avoid any misinterpretation. Cases of atypical adenomatous hyperplasia and metachronous second lung cancers were also excluded. Furthermore, patients were excluded if they were not indicated for curative resection or if there was missing data on tumor size, lymph node metastasis, volume of blood loss, duration of hospital stay, and chest tube placement. The final cohort comprised 2,408 patients. Of these, 2,208 patients presented with no previous extrapulmonary malignancies, and 200 presented with previous extrapulmonary malignancies prior to developing primary lung cancer. The studies involving human participants were reviewed and approved by the Institutional Review Board of the National Taiwan University Hospital, Taipei, Taiwan (approval number: 20200411RIND). Written informed consent was waived owing to the retrospective nature of this study.

Basic characteristics, including age, sex, smoking status, Eastern Cooperative Oncology Group (ECOG) performance status, a family history of lung cancer, underlying comorbidities (diabetes mellitus, hypertension, heart disease, and end-stage renal disease), and preoperative findings (serum carcinoembryonic antigen [CEA] level and pulmonary function test) were retrieved from electronic medical records. An abnormal CEA level was defined as ≥5 ng/mL. The surgical method was classified as sublobar resection (wedge resection and segmentectomy) or extensive resection (lobectomy, bilobectomy, and pneumonectomy). The surgical method was determined at the surgeons' discretion and was approved during a weekly multidisciplinary lung cancer meeting.

Patients were monitored in the outpatient clinic using physical examinations, serum CEA measurements, and chest computed tomography (CT) every 6 months for the first 2 years. Thereafter, patients were monitored using similar methods every 6–12 months, according to the physicians' instructions. Brain magnetic resonance imaging/CT, positron emission tomography/bone scan, bronchoscopy, lymph node biopsy, chest ultrasonography, and other tests were performed whenever any symptoms or signs of tumor recurrence were observed.

### Clinicopathological Features

Information on the tumor size, predominant histological type, degree of differentiation, visceral pleural invasion (VPI), lymphovascular invasion (LVI), pathological T and N stage, number of dissected lymph nodes, number of dissected lymph node stations, and resection margins were collected from pathological reports of preoperative biopsies, intraoperative frozen sections, or postoperative specimens. Malignancies were classified as second primaries when the histological features were distinct or confirmed to be different after immunohistochemical staining. Histopathological patterns were classified according to the 2015 World Health Organization criteria (13). Lung cancer, breast cancer, and thyroid cancer staging were determined based on the eighth edition of the American Joint Committee on Cancer Tumor-Node-Metastasis staging system (14–16).

### Subgroup Analysis of Patients With Primary Lung Cancer With Previous Extrapulmonary Malignancies

The time interval between the diagnosis of the two cancers was defined as the time in years between the diagnosis of a previous extrapulmonary malignancy and that of primary lung cancer. When multiple extrapulmonary malignancies were present in one patient, the shortest time interval was adopted.

Extrapulmonary malignancies were divided into seven subgroups: breast cancer, gastrointestinal cancer, thyroid cancer, gynecological cancer, head and neck cancer, genitourinary cancer, and others. Gastrointestinal cancers included colorectal, gastric, pancreatic, gallbladder, and liver cancers; gynecological cancers included endometrial, cervical, and ovarian cancers; genitourinary cancers included renal, urothelial, bladder, and prostate cancers; and others included hematological, central nervous system, soft tissue, and salivary gland cancers. Patients were defined as having multiple previous extrapulmonary malignancies if they had cancer in more than one of these subgroups.

### Statistical Analysis

Descriptive statistics are reported as mean ± standard deviation for continuous data and as numbers (percentages) for categorical data. Student's *t* test was conducted for continuous variables, and the Chi-square test or Fisher's exact test was conducted for categorical variables, depending on the cell size. The Kaplan–Meier method was used to plot survival curves. Statistical comparisons between survival distributions were made using the log-rank test. Multivariate analysis using the Cox proportional hazards model was performed to identify factors associated with 5-year overall survival (OS). All *p* values were two-sided, and a *p* < 0.05 was considered statistically significant. The statistical software used for all analyses was SPSS for MAC (version 25.0; SPSS, Chicago, IL, USA).

## Results

### Patient Demographics and Clinicopathological Features

The study cohort included 2,408 patients with primary lung cancer, of whom 200 (8.3%) had previous extrapulmonary malignancies, and 59 (2.4%) received neoadjuvant therapy. The mean follow-up time was 39.1 ± 23.4 months. Only age, sex, a family history of lung cancer, and preoperative CEA levels were different between the two groups. Patients with previous extrapulmonary malignancies were older (62.0 ± 11.3 vs. 60.3 ± 11.2 years; *p* = 0.045), predominantly female (71.0 vs. 63.3%; *p* = 0.030), less likely to have a family history of lung cancer (12.5 *vs*. 19.7%; *p* = 0.013), and more likely to have higher preoperative CEA levels (≥ 5 ng/mL: 17.0 vs. 11.0%; *p* = 0.020) than those with lung cancer only. Patient demographics and clinical characteristics are presented in Table 1.

**Table 1:** Demographic and clinical features.

**Feature**	**All**	**Lung cancer only**	**Lung cancer with PM**	***P*-Value**
	**(*n* = 2,408)**	**(*n* = 2,208)**	**(*n* = 200)**	
Age, years	60.4 ± 11.2	60.3 ± 11.2	62.0 ± 11.3	0.045
Sex (female)	1,540 (64.0)	1,398 (63.3)	142 (71.0)	0.030
Smoking	413 (17.2)	381 (17.3)	32 (16.0)	0.652
ECOG PS				0.210
0	1,865 (77.5)	1,703 (77.1)	162 (81.0)	
≥ 1	543 (22.5)	505 (22.9)	38 (19.0)	
PFT				
FVC, % ^a^	107.7 ± 15.7	107.6 ± 15.8	108.7 ± 15.2	0.349
FEV1, % ^a^	107.7 ± 18.8	107.6 ± 18.7	109.0 ± 19.7	0.327
Family history of lung cancer	461 (19.1)	436 (19.7)	25 (12.5)	0.013
Comorbidities				0.737
Yes	968 (40.4)	885 (40.3)	83 (41.5)	
No	1,429 (59.6)	1,312 (59.7)	117 (58.5)	
CEA level ^a^				0.020
Normal	1,905 (88.5)	1,763 (89.0)	142 (83.0)	
Abnormal	248 (11.5)	219 (11.0)	29 (17.0)	

a *Missing values for FVC (n = 126), FEV1 (n = 128), and CEA level (n = 255)*.

*Data are presented as mean ± standard deviation or number (percentage)*.

*CEA, carcinoembryonic antigen; ECOG PS, Eastern Cooperative Oncology Group performance status; FEV1, forced expiratory volume in 1 s; FVC, forced vital capacity; PFT, pulmonary function test; PM, previous extrapulmonary malignancy*.

No significant differences were observed in the pathological features of lung cancer between patients with and without previous extrapulmonary malignancies. The most common histological type in both groups was adenocarcinoma (lung cancer only: 91.5%; lung cancer with previous extrapulmonary malignancies: 88.5%). Among patients with lung cancer with previous extrapulmonary malignancies, 162 (81.0%) had tumors ≤ 3 cm (T1). Detailed information on the pathological features is listed in Table 2.

**Table 2:** Pathological features of the excised lung tumors.

**Feature**	**All**	**Lung cancer only**	**Lung cancer with PM**	***P*-Value**
	**(*n* = 2,408)**	**(*n* = 2,208)**	**(*n* = 200)**	
Histology				0.319
Adenocarcinoma	2,198 (91.3)	2,021 (91.5)	177 (88.5)	
SqCC	111 (4.6)	101 (4.6)	10 (5.0)	
SmCC	1 (0.0)	1 (0.0)	0 (0.0)	
Others ^a^	98 (4.1)	85 (3.8)	13 (6.5)	
Differentiation ^b^				0.052
Well	771 (32.0)	711 (32.2)	60 (30.0)	
Moderate	1,216 (50.5)	1,117 (50.6)	99 (49.5)	
Poor	319 (13.2)	294 (13.3)	25 (12.5)	
VPI+	453 (18.8)	416 (18.8)	37 (18.5)	0.906
LVI+	416 (17.3)	383 (17.3)	33 (16.5)	0.762
Tumor size (cm)				0.795
≤ 3	1,947 (80.9)	1,785 (80.8)	162 (81.0)	
3–5	372 (15.4)	339 (15.4)	33 (16.5)	
5–7	68 (2.8)	64 (2.9)	4 (2.0)	
> 7	21 (0.9)	20 (0.9)	1 (0.5)	
N stage				0.377
N0	2,105 (87.4)	1,933 (87.5)	172 (86.0)	
N1	95 (3.9)	89 (4.0)	6 (3.0)	
N2	208 (8.6)	186 (8.4)	22 (11.0)	
Pathological stage				0.412
0	69 (2.9)	66 (3.0)	3 (1.5)	
IA	1,532 (63.6)	1,400 (63.4)	132 (66.0)	
IB	122 (5.1)	113 (5.1)	9 (4.5)	
II	420 (17.4)	392 (17.8)	28 (14.0)	
III	228 (9.5)	204 (9.2)	24 (12.0)	
IV	37 (1.5)	33 (1.5)	4 (2.0)	
Resection margin ^b^				0.705
Negative	2,237 (94.0)	2,055 (94.0)	182 (93.3)	
Positive	144 (6.0)	131 (6.0)	13 (6.7)	

a *Others included carcinoid tumor (n = 19), invasive mucinous carcinoma (n = 15), lymphoepithelial-like carcinoma (n = 11), pleomorphic carcinoma (n = 9), adenosquamous carcinoma (n = 5), sarcomatoid carcinoma (n = 5), large cell carcinoma (n = 5), and unspecified (n = 23)*.

b *Missing values for differentiation (n = 102) and resection margin (n = 27)*.

*Data are presented as number (percentage)*.

*LVI, lymphovascular invasion; PM, previous extrapulmonary malignancy; SmCC, small cell carcinoma; SqCC, squamous cell carcinoma; VPI, visceral pleural invasion*.

### Perioperative Outcomes

No significant differences in perioperative outcomes were observed between patients with and without previous extrapulmonary malignancies. Sublobar resection was slightly more common than extensive resection in both groups. This may be because most lung cancers were early-stage cancers. Of the 2,408 patients, only two (0.1%) died during the first 30 postoperative days. Other perioperative outcomes are presented in Table 3.

**Table 3:** Perioperative outcomes.

**Factor**	**All**	**Lung cancer only**	**Lung cancer with PM**	***P-*Value**
	**(*n* = 2,408)**	**(*n* = 2,208)**	**(*n* = 200)**	
Surgical method				0.714
Sublobar resection ^a^	1,282 (53.2)	1,178 (53.4)	104 (52.0)	
Extensive resection ^b^	1,126 (46.8)	1,030 (46.6)	96 (48.0)	
Surgical approach				0.416
Thoracotomy	14 (0.6)	12 (0.5)	2 (1.0)	
VATS	2,394 (99.4)	2,196 (99.5)	198 (99.0)	
Non-intubated anesthesia	798 (33.1)	731 (33.1)	67 (33.5)	0.910
Mean number of dissected LNs	9.6 ± 8.2	9.7 ± 8.2	8.9 ± 7.9	0.164
Mean number of dissected LN stations	3.5 ± 1.6	3.5 ± 1.6	3.4 ± 1.7	0.147
Operative bleeding, mL	29.1 ± 108.4	29.1 ± 109.5	29.25 ± 95.6	0.986
Postoperative ICU stay, days	0.3 ± 1.2	0.3 ± 1.2	0.3 ± 0.6	0.669
Postoperative hospital stay, days	4.8 ± 4.1	4.8 ± 4.2	5.1 ± 3.1	0.354
Chest tube duration, days	2.3 ± 2.3	2.3 ± 2.3	2.5 ± 2.4	0.209
Conversion	12 (0.5)	12 (0.5)	0 (0.0)	0.296
30-day mortality	2 (0.1)	2 (0.1)	0 (0.0)	0.670

a *Sublobar resection included wedge resection and segmentectomy*.

b *Extensive resection included lobectomy, bilobectomy, and pneumonectomy*.

*Data are presented as mean ± standard deviation or number (percentage)*.

*ICU, intensive care unit; LN, lymph node; PM, previous extrapulmonary malignancy; VATS, video-assisted thoracoscopic surgery*.

### Correlation Between Clinicopathological Features and Survival

In the univariate analysis, many factors were significantly associated with poor OS, including older age, male sex, poor performance status (ECOG ≥ 1), smoking status, higher preoperative CEA levels (≥ 5 ng/mL), extensive resection, thoracotomy, non-adenocarcinoma histology, poorly differentiated tumors, VPI, LVI, positive resection margins, more advanced stage (stage II–IV), and type of primary malignancy other than breast or thyroid cancer. However, only poor performance status (ECOG ≥ 1), higher preoperative CEA levels (≥ 5 ng/mL), poorly differentiated tumors, more advanced stage (stage II–IV), and type of primary malignancy other than breast or thyroid cancer were independent factors significantly associated with poor OS in the multivariate analysis (Table 4).

**Table 4:** Univariate and multivariate analyses of clinicopathological features and overall survival.

**Variable**	**Univariate analysis**			**Multivariate analysis**		
	**Patients (*n* = 2,408)**	**Number of deaths [5-Year OS (%)]**	***P*-Value**	**HR**	**95% CI**	***P*-Value**
Age, years			<0.001			0.109
≤ 65	1,604	46 (95.4)		1.000		
>65	804	52 (90.2)		1.509	0.913–2.494	
Sex			<0.001			0.214
Female	1,540	42 (95.9)		1.000		
Male	868	56 (89.9)		1.448	0.808–2.598	
ECOG PS			<0.001			0.036
0	1,865	46 (95.8)		1.000		
≥1	543	52 (88.6)		1.716	1.035–2.844	
Smoking status			<0.001			0.956
Never	1,995	68 (94.6)		1.000		
Current or former	413	30 (88.9)		1.018	0.531–1.954	
CEA level^a^			<0.001			0.001
Normal	1,905	51 (95.6)		1.000		
Abnormal	248	20 (85.3)		2.544	1.449–4.464	
Surgical method			0.007			0.148
Sublobar resection^b^	1,282	33 (96.2)		1.000		
Extensive resection^c^	1,126	65 (91.7)		1.513	0.863–2.653	
Surgical approach			<0.001			0.161
Thoracotomy	14	3 (74.1)		1.000		
VATS	2,394	95 (93.8)		0.215	0.025–1.842	
Histological type			<0.001			0.289
Adenocarcinoma	2,198	73 (94.7)		0.690	0.348–1.370	
Non-adenocarcinoma	210	25 (83.3)		1.000		
Differentiation^a^			<0.001			<0.001
Well and moderate	1,987	52 (96.0)		1.000		
Poor	319	39 (76.3)		2.907	1.675–5.047	
VPI			<0.001			0.696
Negative	1,955	46 (96.3)		1.000		
Positive	453	52 (83.7)		1.137	0.597–2.166	
LVI			<0.001			0.054
Negative	1,992	48 (96.1)		1.000		
Positive	416	50 (81.5)		1.706	0.991–2.935	
Resection margin^a^			<0.001			0.578
Negative	2,237	82 (94.4)		1.000		
Positive	144	14 (83.0)		1.303	0.513–3.306	
Stage			<0.001			<0.001
0–1	1,723	24 (97.6)		0.191	0.087–0.421	
2–4	685	74 (84.3)		1.000		
First malignancy			<0.001			
Lung cancer without PM	2,208	82 (94.3)		1.000		
Other cancers as PM	121	16 (81.1)		2.863	1.425–5.753	0.003
Breast or thyroid cancer as PM	79	0 (100.0)		0.000		0.970

a *Missing values for CEA level (n = 255), differentiation (n = 102), and resection margin (n = 27)*.

b *Sublobar resection included wedge resection and segmentectomy*.

c *Extensive resection included lobectomy, bilobectomy, and pneumonectomy*.

*CEA, carcinoembryonic antigen; CI, confidence interval; ECOG PS, Eastern Cooperative Oncology Group performance status; HR, hazard ratio; LVI, lymphovascular invasion; OS, overall survival; PM, previous extrapulmonary malignancy; VATS, video-assisted thoracoscopic surgery; VPI, visceral pleural invasion*.

**Figure 1:**
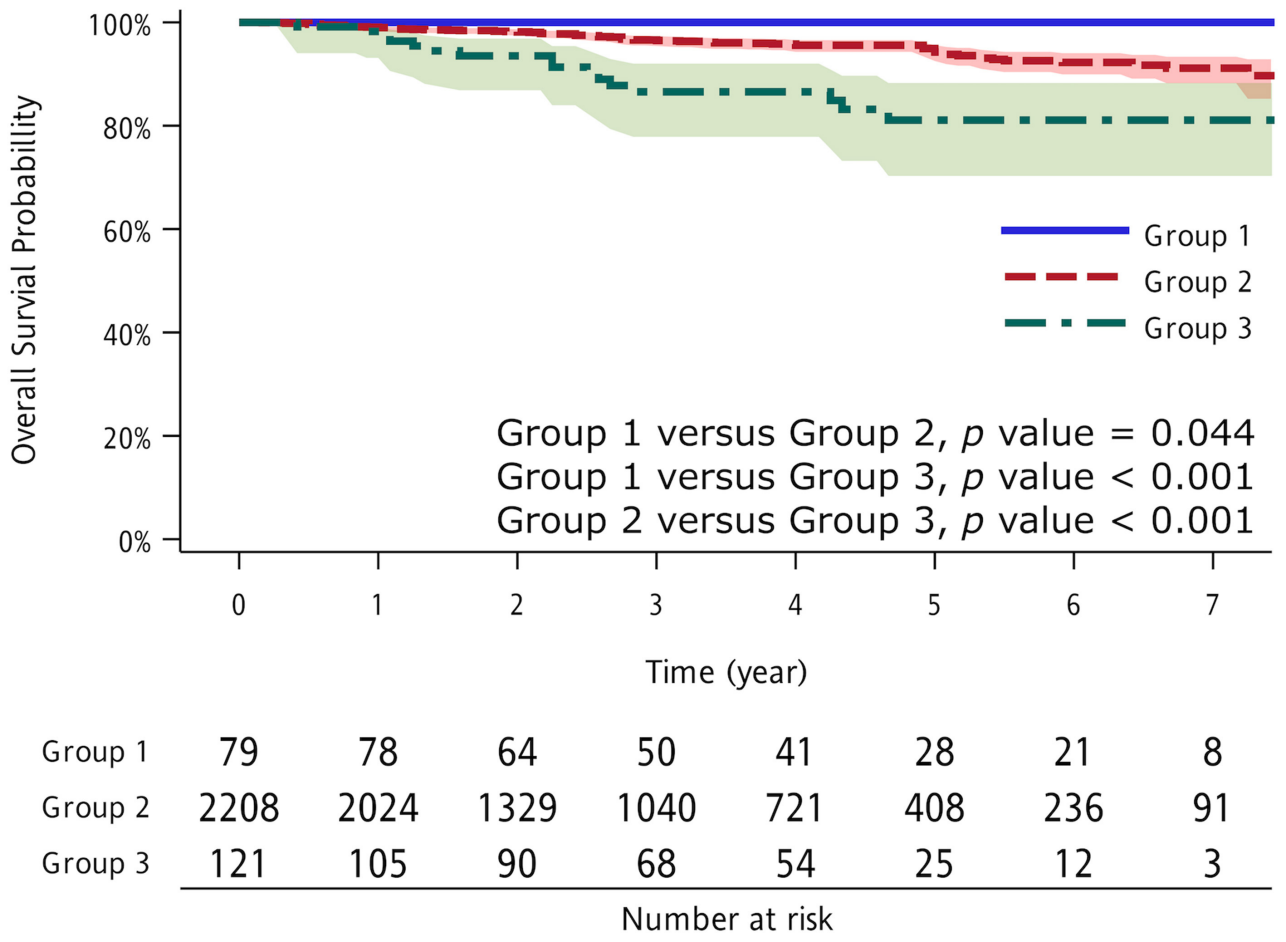
Kaplan–Meier overall survival (OS) curve according to study group. Group 1: thyroid and breast cancer as previous extrapulmonary malignancies; Group 2: no previous extrapulmonary malignancies; and Group 3: other cancers as previous extrapulmonary malignancies.

There was a significant difference in OS between patients with and without previous extrapulmonary malignancies. The 5-year OS in patients with lung cancer only was 94.3%, whereas it was 88.8% in patients with previous extrapulmonary malignancies (*p* = 0.032). After excluding patients with previous breast or thyroid cancer, the difference in 5-year OS between patients with lung cancer only and those with previous extrapulmonary malignancies was even larger (94.3 vs. 81.1%; *p* < 0.001), as no patients with previous breast or thyroid cancer died during follow-up, and previous breast and thyroid cancers were independent factors associated with better OS (Figure 1).

### Clinicopathological Features of Patients With Lung Cancer With Previous Extrapulmonary Malignancies

**Figure 2:**
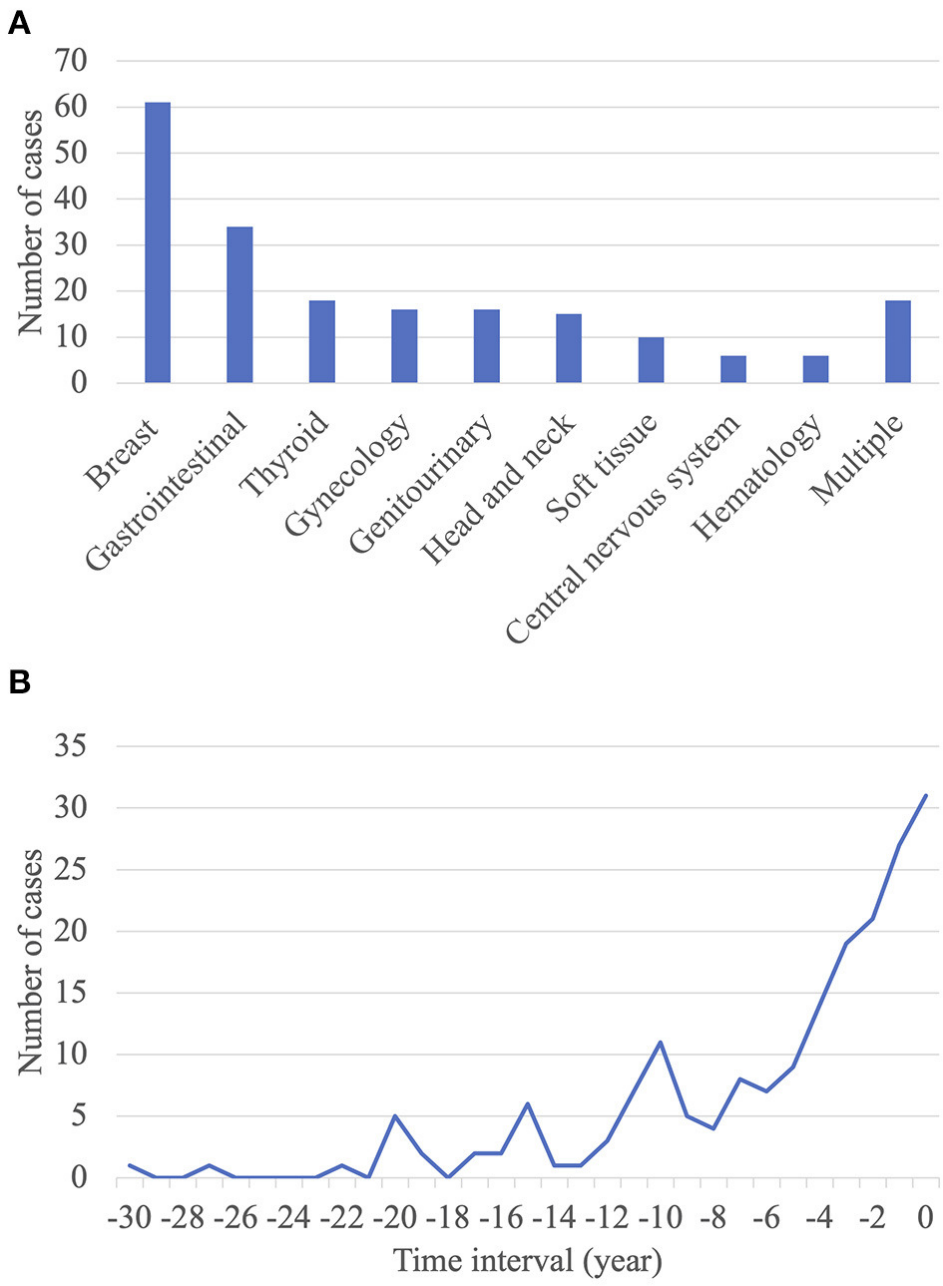
Characteristics of extrapulmonary malignancies. **(A)** Distribution of occurrence sites of previous extrapulmonary malignancies. **(B)** Time interval between the diagnosis of previous extrapulmonary malignancies and that of primary lung cancer.

Among the 200 patients with lung cancer with previous extrapulmonary malignancies, breast, gastrointestinal, and thyroid cancers were the most frequent previous extrapulmonary malignancies. Of the 34 patients with gastrointestinal cancer, 20 had colorectal cancer, six had gastric cancer, five had liver cancer, one had pancreatic cancer, one had gallbladder cancer, and one had a gastrointestinal stromal tumor (Figure 2A). As stated above, previous breast or thyroid cancer was associated with improved survival compared to other previous extrapulmonary malignancies. There were only two patients with stage IV lung cancer and zero pneumonectomy or bilobectomy in this group. Furthermore, by analyzing the available data on the staging of previous extrapulmonary malignancies, majority of the patients present with early breast and thyroid cancer. Conversely, previous gastrointestinal, head and neck, or gynecological cancer was associated with poor survival, with hazard ratios of 3.162 (*p* = 0.025), 5.017 (*p* = 0.006), and 6.162 (*p* < 0.001), respectively (Supplementary Figure 1; Supplementary Tables 1, 3).

The mean interval between the diagnosis of a previous extrapulmonary malignancy and that of primary lung cancer was 5.4 ± 5.8 years. Approximately half of the patients (*n* = 112; 56.0%) had an interval of ≤ 5 years (Figure 2B). There was no significant correlation between the interval between tumor diagnoses and OS (Supplementary Table 1).

## Discussion

Lung cancer is the leading cause of cancer mortality in the United States and worldwide (17). The outcomes of patients with lung cancer with previous extrapulmonary malignancies have received much attention in recent decades. However, the impact of previous malignancies on survival and prognosis is still not clearly defined. Our study showed that the incidence of previous extrapulmonary malignancies was 8.3% in patients with primary lung cancer. Several clinicopathological features, including age, sex, a family history of lung cancer, and preoperative CEA levels, were significantly different between the two groups. Furthermore, patients with previous breast or thyroid cancer had significantly better OS than those without previous malignancies. Conversely, patients with other previous extrapulmonary malignancies had significantly poorer OS.

The incidence of previous extrapulmonary malignancies in our study was similar to those reported previously (1–22%) (7, 8, 18–26). Corresponding to an increase in the incidence of multiple primary cancers, a surge of 5.7% in lung cancer as a second primary malignancy was also noted between 1988–1992 and 2011–2014 in the United States National Cancer Institute's Surveillance, Epidemiology, and End Results Program (19). Aside from common risk factors, such as cancer-promoting lifestyle factors and environmental interactions (27), genetic predisposition in the form of germline mutations (28, 29) and the subsequent carcinogenic effects of cancer treatment have been reported as potential mechanisms for the development of multiple primary cancers. Additional lung cancer risk was observed in patients with Hodgkin's lymphoma who were treated with radiotherapy (30, 31). A meta-analysis of 13 studies demonstrated that radiotherapy for breast cancer was significantly associated with a relative risk of 1.22 for second lung cancer (32), with an excess relative risk of 8.5% per Gray (33). Another well-established explanation for the growing incidence of multiple primary tumors is the prolonged survival rates of patients with cancer combined with increased concern over personal health and surveillance among cancer survivors (8).

Supplementary Table 2 reviews several studies that examined clinicopathological features in patients with lung cancer with previous extrapulmonary malignancies. Some reported that patients with previous extrapulmonary malignancies were older (18, 20, 23, 24). We found similar results; in our study, these patients were ~2 years older than those without previous extrapulmonary malignancies. This may be attributable to the longer exposure to carcinogens and greater genetic susceptibility in older individuals (34). These patients also had higher preoperative CEA levels. This marker underlies a distinct characteristic of extra- or intrapulmonary adenocarcinoma among patients with multiple primary malignancies (35). However, smoking habits between the two study subgroups did not differ, which agrees with the mathematical modeling of the Liverpool Lung Project (LLP). By showing a higher impact of cumulative non-smoking risk on lung cancer over smoking alone, additive effects of lung disease and environmental exposures should be recognized in the non-smoking population (36). Another significant feature that contradicted with accepted knowledge is the lower incidence of a family history of lung cancer. This may indicate a different etiology in this subgroup, corresponding with those of previous studies suggesting that the occurrence of multiple malignancies may be multifactorial in origin (20–23, 26). Further studies are warranted to investigate the hereditary contribution toward tumorigenesis in this subgroup.

Exposure to prior cancer treatment may lead to treatment intolerance in subsequent cancers. This affects the surgeons' selection of surgical methods and postoperative management. However, our results showed no differences in preoperative pulmonary reserve or intraoperative surgical margins in patients with and without previous extrapulmonary malignancies. Therefore, there was no need for extra consideration of surgical method selection, tumor approach, or preference for non-intubated airway management. Moreover, surgical blood loss, length of postoperative hospital stay, and chest tube duration did not differ between the two groups, indicating that no special care was needed. This result corresponds with those of several previous studies (23, 25).

Because of the undetermined interference caused by the previous cancer, whether to include patients with multiple primary malignancies in clinical trials remains controversial (37–40). Our results indicate that some previous extrapulmonary malignancies have a negative impact on survival. However, it is noteworthy that prior breast or thyroid cancer resulted in a better outcome. Higher performance status, lower CEA levels, well and moderately differentiated tumors, and early-stage cancers also served as favorable prognostic factors in the multivariate analysis. Broader inclusion of different subgroups of patients with lung cancer in future clinical trials is necessary to expand their authenticity and generalizability. The factors we identified may serve as the eligibility criteria to confirm equality between patients with and without previous extrapulmonary malignancies. Notably, in our study, the interval between the diagnoses of the two cancers was concentrated in the first 5 years and did not significantly impact survival trends. This calls into question the legitimacy of the current practice of excluding prior cancer within 5 years of enrollment and underscores the concerns of excluding specific groups of patients.

Breast cancer was the most common previous extrapulmonary malignancy reported by our and other studies (7, 8, 19, 20, 22, 24, 25). This may have accounted for the higher proportion of female patients in our group with lung cancer with previous extrapulmonary malignancies. According to the Taiwan Cancer Registry, breast and lung cancer have been among the top three most common cancers since 2003. The launch of a national screening program for breast cancer (41) and the recent advocation of low-dose CT (42) have been reported as factors contributing to this phenomenon, leading to greater discovery of both cancers.

Our results should be interpreted in light of several limitations. First, the study cohort only consisted of surgically treated patients. This may have underestimated the overall incidence of lung cancer in patients with previous extrapulmonary malignancies. Nevertheless, as the largest tertiary hospital in Taiwan, one-third of the early lung cancer resections in the country are performed at our institution, which provides an adequate number of samples. Second, as a single-center, retrospective study, our results may not be generalizable to the entire population. Although further studies are required in patients of different ethnic backgrounds, we reduce other possible bias by conducting our research over a short period of time and treat our patients under similar operative protocol. Third, because of the lack of data linkage between hospitals, we are incapable of knowing the status of previous malignancies, if they were evaluated and treated outside our hospital. However, by analyzing the existing documentation in the electronic medical record, we did make an effort to explore the additional relationships between previous cancer staging and lung cancer outcome.

In conclusion, lung cancer was more likely to develop in patients with previous breast, gastrointestinal, or thyroid cancer within 5 years after the first primary malignancy diagnosis. Although OS was lower in patients with previous extrapulmonary malignancies, previous breast or thyroid cancer did not increase mortality. Our findings may help surgeons to predict the prognosis in this subgroup of patients with primary lung cancer, and to arrange follow-up examinations more frequently.

## Data Availability Statement

The raw data supporting the conclusions of this article will be made available by the authors, without undue reservation.

## Author Contributions

M-WL, H-HH, J-SC, and M-SH contributed to conception and design of the study. J-HC, H-YL, P-HC, and X-HC organized the database. K-CT, T-MT, and H-CL performed the statistical analysis. H-YL wrote the first draft of the manuscript. X-HC, P-HC, K-CT, and M-WL wrote sections of the manuscript. All authors contributed to manuscript revision, read, and approved the submitted version.

## Funding

The authors declare that this study received funding from the Ministry of Science and Technology, Taiwan [grant number: 107-2221-E-002-080-MY3], National Taiwan University Hospital, Taiwan [grant number: NTUH109-S4659] and Taiwan Lung Foundation. The funder had the following involvement with the study: English language editing and article processing fee. The funder was not involved in the study design, collection, analysis, interpretation of data, the writing of this article, or the decision to submit it for publication.

## Conflict of Interest

The authors declare that the research was conducted in the absence of any commercial or financial relationships that could be construed as a potential conflict of interest.

## Publisher's Note

All claims expressed in this article are solely those of the authors and do not necessarily represent those of their affiliated organizations, or those of the publisher, the editors and the reviewers. Any product that may be evaluated in this article, or claim that may be made by its manufacturer, is not guaranteed or endorsed by the publisher.
