# Supplementary material for: Previous Extrapulmonary Malignancies Impact Outcomes in Patients With Surgically Resected Lung Cancer
Source: Front Surg. 2021 Oct 5;8:747249. doi: 10.3389/fsurg.2021.747249 (PMC8523860; doi:10.3389/fsurg.2021.747249)
Supplement: Supplementary file 1 [file Table_1.DOCX]

**Supplementary Table 1**

Correlation between clinicopathologic features and survival in lung cancer with previous extrapulmonary malignancy

| Variables | Patient no. | HR | 95% CI | *p* value |
| --- | --- | --- | --- | --- |
| Occurrence location |  |  |  |  |
| Lung cancer without PM | 2208 | 1.000 |  |  |
| Breast | 61 | 0.000 |  |  |
| Thyroid | 18 | 0.000 |  |  |
| Multiple | 18 | 1.212 | 0.169-8.716 | 0.848 |
| Others | 22 | 2.277 | 0.560-9.263 | 0.251 |
| Genitourinary | 16 | 2.567 | 0.630-10.454 | 0.188 |
| Gastrointestinal | 34 | 3.162 | 1.158-8.636 | 0.025 |
| Head and neck | 15 | 5.017 | 1.583-15.898 | 0.006 |
| Gynecology | 16 | 6.162 | 2.257-16.821 | <0.001 |
| Interval between occurrences (year)^a^ |  |  |  |  |
| <1 | 31 | 1.000 |  |  |
| 1≤ and <5 | 81 | 0.661 | 0.193-2.261 | 0.509 |
| 5≤ and <10 | 33 | 0.205 | 0.023-1.831 | 0.156 |
| ≥10 | 43 | 0.587 | 0.131-2.631 | 0.487 |

^a^Missing value from interval (n=12)

Abbreviations: CI confidence interval, HR hazard ratio, No number, PM previous extrapulmonary malignancy

**Supplementary Table 2**

Summary of existing studies on lung cancer patients with previous extrapulmonary malignancy

|  | **Published year/ Study period** | **Cases identified** | **Common previous malignancies** | **Characteristic features of patients with previous malignancies** | **Differences in overall survival** |
| --- | --- | --- | --- | --- | --- |
| Current Study | 2011-2017 | 200/2408 (8.3%) | Breast (30.5%), gastrointestinal (17%), thyroid (9%) | Older age, women, family history of lung cancer, abnormal CEA level | Superior in patients with breast or thyroid cancer as previous malignancies; inferior in patients with other cancers as previous malignancies |
| Hu et al.^8^ | 2014-2018 | 178/3790 (5.0%) | Breast (20%), colorectal (16%) | Women, suspected detection in health examination, smaller size, earlier stage | Not significant |
|  |  |  |  |  |  |
| Deng et al.^19^ | 1988-2014 | 85,758/765,299 (11.2%) | Prostate (24.1%), breast (15.9%), colon and rectum (11.4%), urinary bladder (9.9%) | Diagnosed at localized stage, smaller primary tumor, treated with surgery | Lower risk of lung cancer-specific and overall mortality in the first 5 years but higher risk thereafter |
| Lu et al.^21^ | 2004~2012 | 99/1611 (6.15%) | Oropharynx (20%), colon (16%), gynecological (12%), uroepithelial (11%) | Earlier stage, surgical treatment, less supportive treatment | Best in patients with second malignancy at follow-up, then in patients with synchronous malignancy, and worst in patients with pre-existing malignancy |
| Shan et al.^20^ | 2000-2010 | 283/27642 (1.02%) | Colorectal (22.0%), breast (18.4%), gastric (14.4%), larynx (11.9%) | Women, older age, squamous cell histology, less small cell histology, earlier stage, family history of cancer | Not significant |
| Pagès et al^23a^ | 1980-2009 | 1243/5846 (22%) | Lung (6.7%), head and neck (6.7%) | Older age, women, earlier stage, less induction, fewer pneumonectomies | Inferior in patients with previous malignancies |
| Pagès et al.^24^ | 1980-2009 | 389/4992 (8%) | Bladder (13.4%), colorectal (16%), prostate (15%), breast (19%) | Women, older age, non-smokers, ADC, smaller size, second nodule in the same lobe | Not significant |
| Donin et al.^18^ | 1992-2008 | 25,472/1,450,837 (1.75%) | Hypopharynx, oropharynx, larynx, tonsil, bladder, esophageal | Men, older age, being divorced/widowed/separated | Not stated |
| Quadrelli et al.^25^ | 1986-2007 | 34/414 (8.2%) | Breast (20.5%), uroepithelial (20.5%), lung (15.4%) | Not stated | Not significant |
| Reinmuth. et al.^22^ | 2004-2006 | 444/3142 (14.1%) | Breast (15.5%), prostate (14.9%), bladder (9.0%), kidney (8.8%) | Current and former smokers tended to have previous cancer of the prostate, gastrointestinal, and head-neck regions | Superior in patients with previous malignancies |
| Furák et al.^26^ | 1997-2006 | 43/911 (4.7%) | Laryngo-pharyngeal (32%), lung (16%), colon (12%) | Not stated | Inferior in patients with previous malignancies |
| Hofmann et al.^7^ | 1996-2005 | 163/5284 (3.0%) | Women: genital (36.4%), breast (22.7%), gastrointestinal (18.2%)  Men: laryngo-pharyngeal (24.1%), gastrointestinal (19.9%), prostate (14.9%). | NSCLC, earlier stage, curative treatment | Superior in patients with previous malignancies |

^a^Include patients with two or more previous malignancies

ADC, adenocarcinoma; CEA, carcinoembryonic antigen; NSCLC, non-small cell lung cancer

**Supplementary Table 3**

TNM staging of breast and thyroid cancer as previous extrapulmonary malignancies.

| TNM stage | Patient no. (%) |
| --- | --- |
| Breast cancer |  |
| Total Patients | 61 |
| 0 | 10 (16.4) |
| I | 13 (21.3) |
| II | 7 (11.5) |
| III | 2 (3.3) |
| IV | 1 (1.6) |
| Missing value | 28 (45.9) |
| Thyroid cancer |  |
| Total Patients | 18 |
| I | 8 (44.4) |
| II | 1 (5.6) |
| III | 0 (0) |
| IV | 0 (0) |
| Missing value | 9 (50) |
