# Supplementary material for: Previous Extrapulmonary Malignancies Impact Outcomes in Patients With Surgically Resected Lung Cancer
Source: Front Surg. 2021 Oct 5;8:747249. doi: 10.3389/fsurg.2021.747249 (PMC8523860; doi:10.3389/fsurg.2021.747249)

Supplementary Figure 1

Kaplan-Meier overall survival curve according to the occurrence location of previous extrapulmonary malignancy.

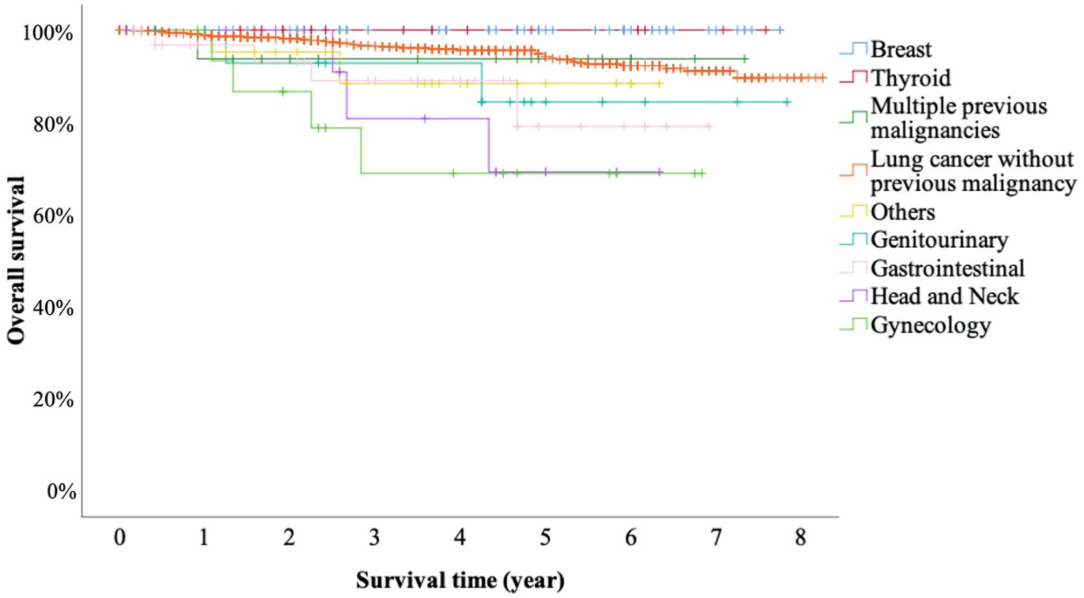

Supplement: Supplementary file 2 [file Image_1.PDF]
